# Detection of Two Pospiviroid Species Affecting Tomato and Pepper Crops in Saudi Arabia

**DOI:** 10.3390/v18091028

**Published:** 2026-09-16

**Authors:** Mahmoud A. Amer, Ammar Haddad, Ibrahim M. Al-Shahwan, Omer Ahmed Abdalla, Muhammad Amir, Khadim Hussain, Mohammad A. Al-Saleh

**Affiliations:** Plant Protection Department, College of Food and Agriculture Sciences, King Saud University, P.O. Box 2460, Riyadh 11451, Saudi Arabia; haddadammar@hotmail.com (A.H.); ibrahim.a173@yahoo.com (I.M.A.-S.); oabdalla55@hotmail.com (O.A.A.); amiraim1122@gmail.com (M.A.); khadim787@gmail.com (K.H.)

**Keywords:** solanaceous, viroids, TASVd, RT-CR, sequence, cultivar response

## Abstract

During the growing season 2018–2019, a total of 199 tomato and 21 pepper asymptomatic and symptomatic samples were collected from different major regions of Saudi Arabia. Using the general Norgen Kit and universal Pospi1-RE/Pospi1-FW primers for Pospiviroid RT-PCR detection, only seven tomato samples and three pepper samples were positive, which amplified the PCR products with the expected sizes of ~364 bp and /~350 bp respectively, confirming the presence of at least one of the nine Pospiviroid species. The PCR amplified products were sent for sequencing, and the results confirmed the presence of tomato apical stunt viroid (TASVd) and pepper chat fruit viroid (PCFVd). However, further confirmation was conducted using specific primers for TASVd and PCFVd that amplify the complete genome. One positive TASVd Saudi isolate was selected to evaluate the severity and symptom responses to mechanical inoculation of four tomato cultivars that are often grown in greenhouses. According to the data obtained, each cultivar exhibited a significant change in each metric. The studied cultivars’ plant heights did not differ significantly from one another. The greatest decrease in vegetative dry weight (39.3%) was observed in the Newton cultivar; with just a 6% decrease in comparison to the healthy controls, the Alindi cultivar was the least impacted. With a 90.2% decrease in fruit output when compared to its untreated control, the Newton cultivar was the most impacted; meanwhile, with a 49.1% decrease in fruit output, the Irit cultivar was the least impacted. The infection of viroids and their economic impact on commercial tomato cultivars have been discussed.

## 1. Introduction

The family *Solanaceae* includes several economically significant crops, like tomatoes (*Solanum lycopersicum*), pepper (*Capsicum* spp.), potatoes (*S. tuberosum*) and eggplant (*S. melongena*), as well as some ornamental plants, such as many species of petunia. It also includes some wild weeds (*S. nigrum*), which grow within or adjacent to cultivated crops and may represent primary reservoirs and sources of infection for viruses and viroids [1,2,3].

In Saudi Arabia, the tomato is a major vegetable crop with significant economic importance, achieving a high self-sufficiency rate of 76% in 2023. It is cultivated in various regions, using both traditional open-field methods and advanced greenhouse technologies to meet year-round demand. The total area under tomato production in Saudi Arabia in 2023 was approximately 15,208 hectares. This area is designated for both open-field and protected agriculture (greenhouse) cultivation. The total quantity of tomatoes produced was over 691,875 tons. According to data from the Saudi Ministry of Environment, Water, and Agriculture in 2023, greenhouse tomato planting accounts for almost 59% of Saudi Arabia’s total tomato fruit production, although it is far smaller than open fields. This illustrates the significance of greenhouse production in contrast to tomato cultivation in open fields. Peppers are also an important crop, but the specific 76% figure applies to tomatoes (The General Authority for Statistics (GASTAT) Publishes 2023 Agricultural Statistics Results).

Unfortunately, greenhouse-grown solanaceous crops are more highly susceptible to viroid infections than those in an open field, which may lead to an epidemic for tomato planting due to important factors like mechanical transmission and infected seeds, and these infections can lead to significant economic losses and potential epidemics [4,5]. So far, no report or study has been conducted in Saudi Arabia about viroid infection in any cultivated crop, except for one report, which described the symptoms of potato spindle tuber viroid (PSTVd) on potato based on symptoms only [6]. This highlights the significance of this study, since it is the first study to provide insight into this emerging area of study in the Kingdom of Saudi Arabia and other Arabian Peninsula countries.

The smallest infectious agent ever known, the viroid, is one of the big threats to these crops, due to its diversity, stability, and the difficulty that is usually experienced in its identification, especially in greenhouse farming [7,8]. Viroids produce a wide range of symptoms, including stunting, leaf/stem chlorosis, epinasty, necrosis and foliar/fruit deformation, dwarfing, vein discoloration, cracking, and delayed growth, depending on host plant cultivars and viroid species [9,10,11].

There are currently 40 viroid species that are members of the Pospiviroidae family, and 5 viroid species that belong to the Avsunviroidae family have been identified, as documented on the ICTV website [12]. Within the family Pospiviroidae, eight species from the genus Pospiviroid have been isolated from naturally infected tomato plants [13,14]. Two viroids have been reported from naturally infected pepper crops, pepper chat fruit viroid (PCFVd) and PSTVd [13,14,15,16,17]. In addition, the citrus exocortis viroid (CEVd) was identified in naturally infected plants of eggplant [18]. Given the lack of data on Solanaceae viroid detection and epidemiology in Saudi Arabia, the primary aim of this research was to identify the presence of the important Pospiviroid infecting tomato and pepper crops. Furthermore, investigations of cultivar response analysis were undertaken against tomato apical stunt viroid (TASVd) in Saudi Arabia.

## 2. Materials and Methods

### 2.1. Field Sample Collection

**Figure 1:**
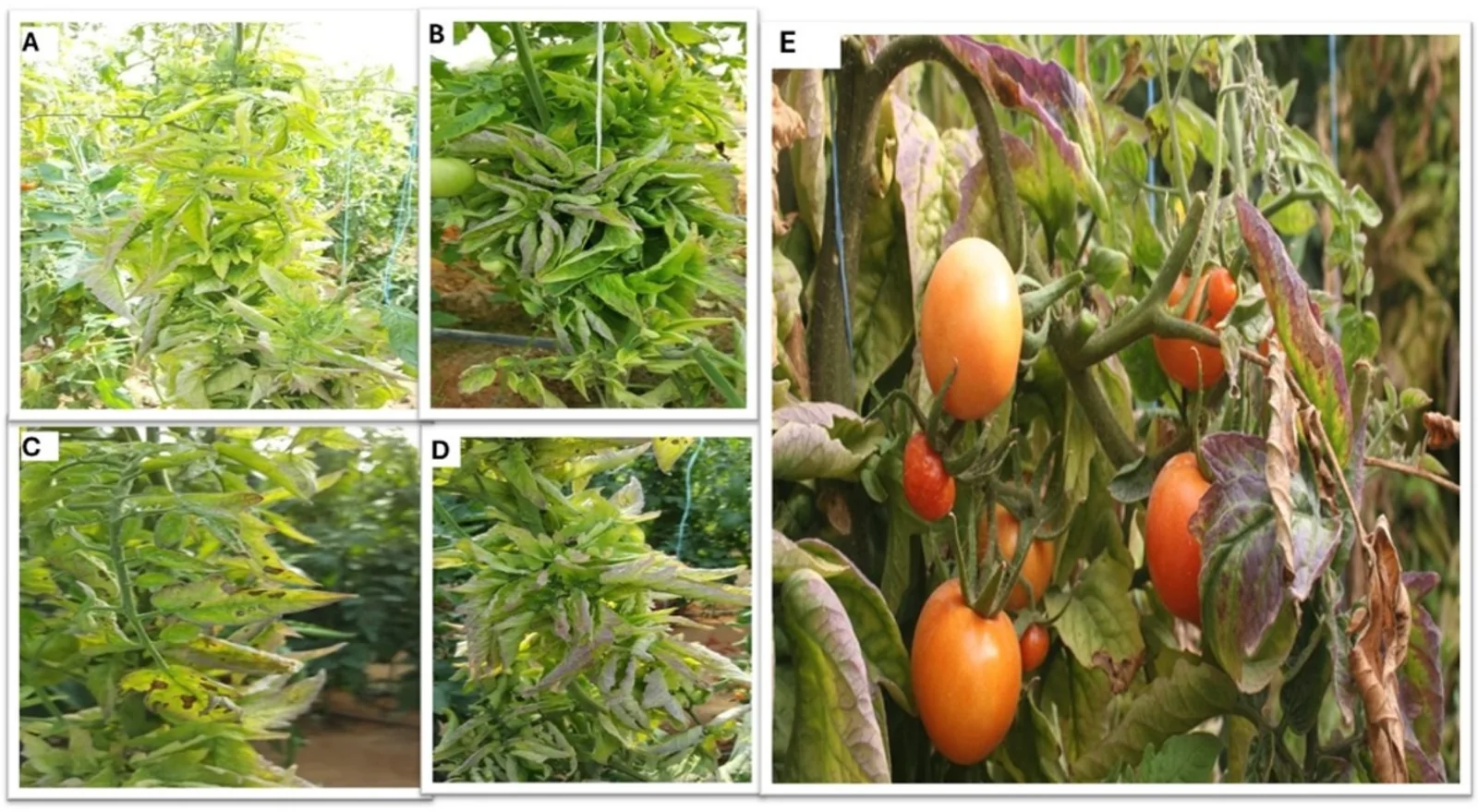
Naturally infected with tomato apical stunt viroid (TASVd) on tomato leaves expiating brightening (**A**), leaf curling (**B**), necrosis (**C**), purple leaf symptoms (**D**) and tomato fruits showing a pale red color, early maturity and reduced fruit size (**E**).

**Figure 2:**
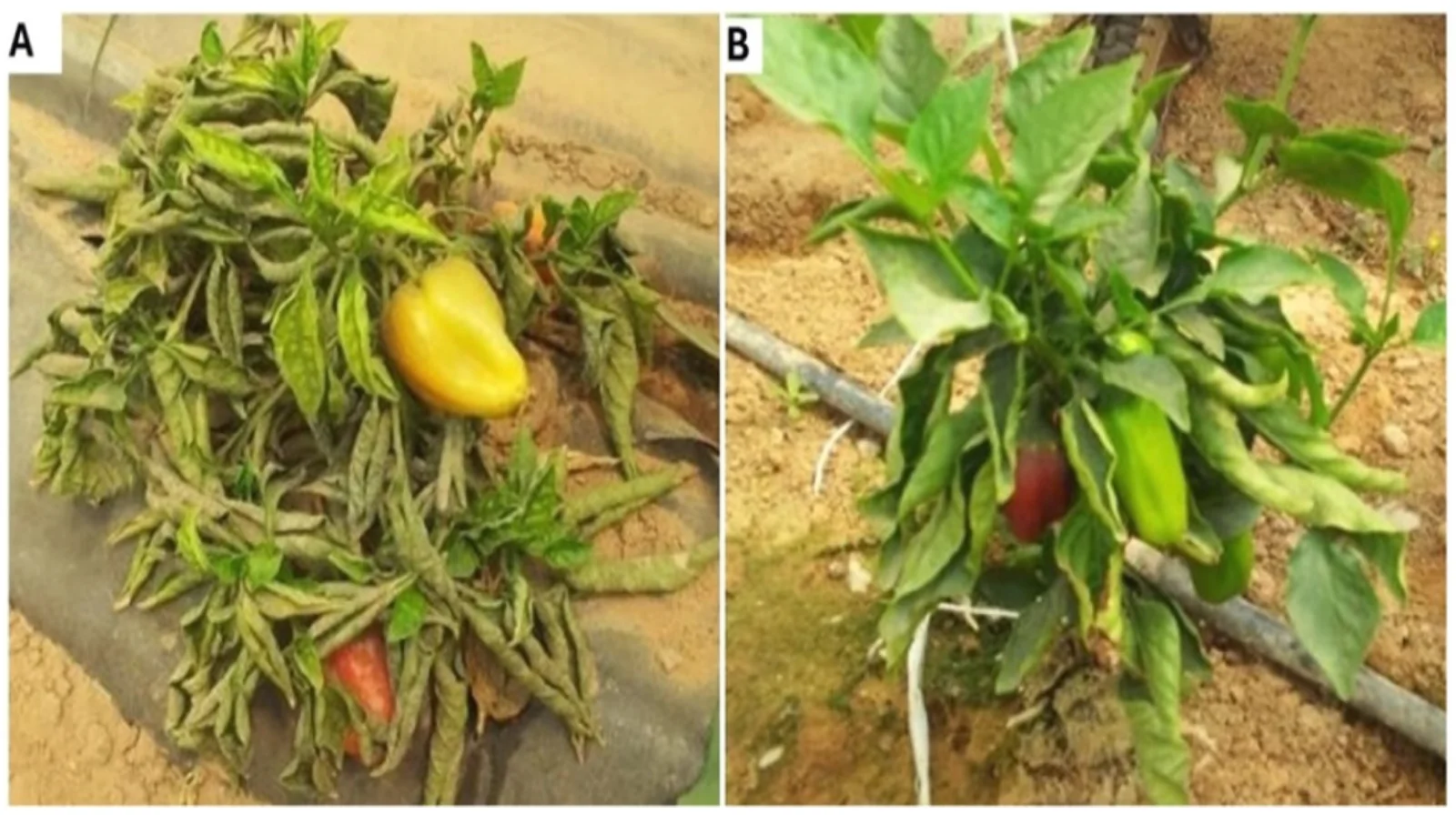
Naturally infected pepper plant in the greenhouse, infected with TASVd, and showing different symptoms, including sever stunting, leaf curling, mottling and small fruits (**A**). Several symptoms on pepper plant infected with PCFVd, which include stunting, leaf curling, mottling and small fruits (**B**).

During the growing survey (2018–2019), a total of 199 tomato samples from different varieties, including Newton (Syngenta), Beisuzhok (Giverech), Alindi (Enza Zaden) and Irit (Rigk Zwan), were collected from symptomatic and asymptomatic plants growing in the five greenhouses of each of the following regions, Riyadh, Al-Qassim, Eastern province, and Tabouk. Moreover, 21 pepper samples from Habhar Shaqra and Rida growing in two greenhouses in Riyadh and Al-Qassim were collected and contained both symptomatic and asymptomatic plants. The symptomatic samples showed viroid-like symptoms, such as stunting, yellowing, leaf curling, witches’ broom and apical stunting, and fruits were smaller and ripped earlier (Figure 1 and Figure 2).

### 2.2. Reverse Transcriptase Polymerase Chain Reaction (RT-PCR)

The total RNA was isolated from the collected samples using an Isolate II RNA Plant Kit, BIO-52077, (Bioline Ltd., London, UK) following the manufacturer’s protocol. All the tomato and pepper plant samples were tested by an RT-PCR assay using general Pospiviroid RT-PCR Detection Kit Product #53700 (Norgen Biotek Corp., Thorold, ON, Canada) following the manufacturer’s instruction to detect all the 9 different viroids that belong to the genus Pospiviroid. The general primers Pospi1-RE/Pospi1-FW [19,20] were used for confirming the occurrence of Pospiviroid, which amplifies the complete genome (~360-bp) product.

Samples that reacted positively with the initial RT-PCR were further assayed for another run of RT-PCR with each of the following specific primers, TASVd (Viroid, Vir+/Vir-) that amplifies a full genome at 360 bp [21], and PCFVd (PCF-seq-F/PCF-seq-R) that amplifies the complete genome at ~350-bp [22,23].

For the RT-PCR test, the BioScript One-Step RT-PCR kit (Bioline Ltd., UK) was used. For each sample, 2 µL of the template RNA was placed in a purified PCR test tube and mixed with 2 µL from each pair of the primers, 2 µL of the enzyme mix, and 25 µL of the PCR buffer. The mixture was then completed to 50 µL with RNAase free distilled water. All test tubes were placed in the Advanced-Primus 96 thermal cycling machine (PEQLAB Ltd., Fareham, UK), which was pre-set at the following thermal cycle protocol: 30 min at 50 °C for the reverse transcriptase, 2 min at 95 °C for initial denaturation, 30 s at 94 °C for denaturation, 30 s at 62 °C for annealing, and 45 s at 72 °C for elongation (steps 3–5 were repeated for 35 cycles, with 10 min at 72 °C for final elongation and hold at 4 °C). Then, 5 µL of the resulting product was loaded on a micro-well of 2% agarose gel prepared in 1x Tris Borate EDTA (TBE) buffer with ethidium bromide. The gel was placed under electrophoresis and allowed to run for 45 min at 120 V and room temp. The PCR Sizer 100 bp DNA Ladder (Norgen Biotek Corp., Thorold, ON, Canada) and 1KB GeneDirex DNA Marker (GeneDireX, Inc., Taoyuan City, Taiwan) were used. The amplified results of cDNA were then visualized, and photos were taken in a gel documentation system.

### 2.3. Sequence and Phylogenetic Analysis

Ten samples that reacted positively with the RT-PCR described earlier using universal primers were purified using the QIAquick Gel Extraction Kit (QIAGEN GmbH, Hilden, Germany) according to the manufacturer’s instructions and directly sequenced in both directions by Sanger sequencing and sent to the “Advanced Genetic Technologies Center” (AGTC), College of Agriculture Sciences, University of Kentucky, USA for complete genome sequencing.

The obtained sequence results were blasted using the National Center for Biotechnology Information (NCBI) after cleaning the noisiness of the obtained data using the BIOEDIT program. Three of our TASVd and one of the PCFVd Saudi isolates were compared with 26 and 21 other worldwide isolates from GenBank for each viroid species. For a better understanding of the analysis results, a phylogenetic tree was constructed to compare the phylogenetic relationship between viroid isolates. Sequences aligned with ClustalW were used to construct a phylogenetic tree using MEGA-X with the Maximum-Likelihood approach and with 1000 bootstrap replications, as implemented by the MEGA 7.0. Sequence Demarcation Tool (SDTv1.3), and MegAlign, as implemented by Lasergene, which were used for pairwise sequence comparisons [24]. The Saudi isolates’ nucleotide identity table was created using DNAStar (19.0) software and compared with other isolates that were reported in the GenBank database.

### 2.4. Cultivar Responses

A greenhouse experiment was conducted to evaluate the responses of selected tomato cultivars to the mechanical inoculation with the Saudi TASVd isolate. Four cultivars were selected, namely, Newton (Syngenta), Beisuzhok (Giverech), Alindi (Enza Zaden) and Irit (Rigk Zwan). The first two were the most grown cultivars in the Alkharj area (one of the most important tomato greenhouses growing locations), and the other two were recently introduced to the Saudi market and were claimed to be Begomovirus-tolerant cultivars. Twenty plants from each cultivar were grown in a controlled greenhouse (at 25–27 °C) for one month before mechanical inoculation. Each cultivar was split into two rows, with 10 pots of each. One row was inoculated treatment, and the other row was inoculated with the buffer as a control.

For mechanical inoculation, 1 g of fresh leaf tissue infected with TASVd isolate was ground in a mortar and pestle with 4 mL of potassium phosphate buffer pH 7.0. The crude sap was rubbed against the pre-dusted leaves of the test plants with ~10 at the true leaf stage to allow the plants to grow for fruit production with Carborundum powder (400 mish), and control plants were inoculated with the potassium buffer only. All plants were kept for 4 months until the most of infected plants died.

For evaluation, all fruits (both mature and immature) were picked at one time at the end of the experiment from each plant and weighed, the plant’s height was recorded and the weight of dry matter of the whole vegetative growth was taken. For vegetative dry weight evaluation, each plant was cut from the vegetative growth above soil level, chopped into smaller pieces, placed in a paper bag and kept in a greenhouse for two weeks until complete dryness. Plants were weighed after two weeks of dryness. Data was taken from each inoculated plant as well as the controlled plants for comparison. All plants that showed symptoms after inoculation were assayed with RT-PCR against TASVd infection as described earlier, to confirm the viroid infection. All data were statistically analyzed using one-way analysis of variance, and differences between control and inoculated plants were measured for each of the indicated parameters with the Least Significant Difference test (LSD) at the 5% significance level; for each parameter, the analysis was performed after the percentages of reduction were transformed to arcsine [25]. The LSD test also was used to separate the means in percent reductions among the different cultivars for the previously mentioned parameters following their inoculation with TASVd isolate.

## 3. Results

### 3.1. Field Observation

Collected samples showed a range of symptoms that vary according to plant species, cultivar, and growth stage. Leaf symptoms of tomato plants that were positively infected with TASVd (according to RT-PCR results later) vary from mild mottling to severe necrosis as well as leaf curling, discoloration, shortened apical internodes, deformation, in some cases brightening and a leaf color turned purple (Figure 1). Leaf samples of pepper plants that were infected with TASVd (according to the RT-PCR assay later) were stunted plants, and leaves showed mottling and leaf curling. Similar symptoms (Figure 2) were associated with plants that reacted positively with PCFVd in the RT-PCR assay.

### 3.2. RT-PCR

**Figure 3:**
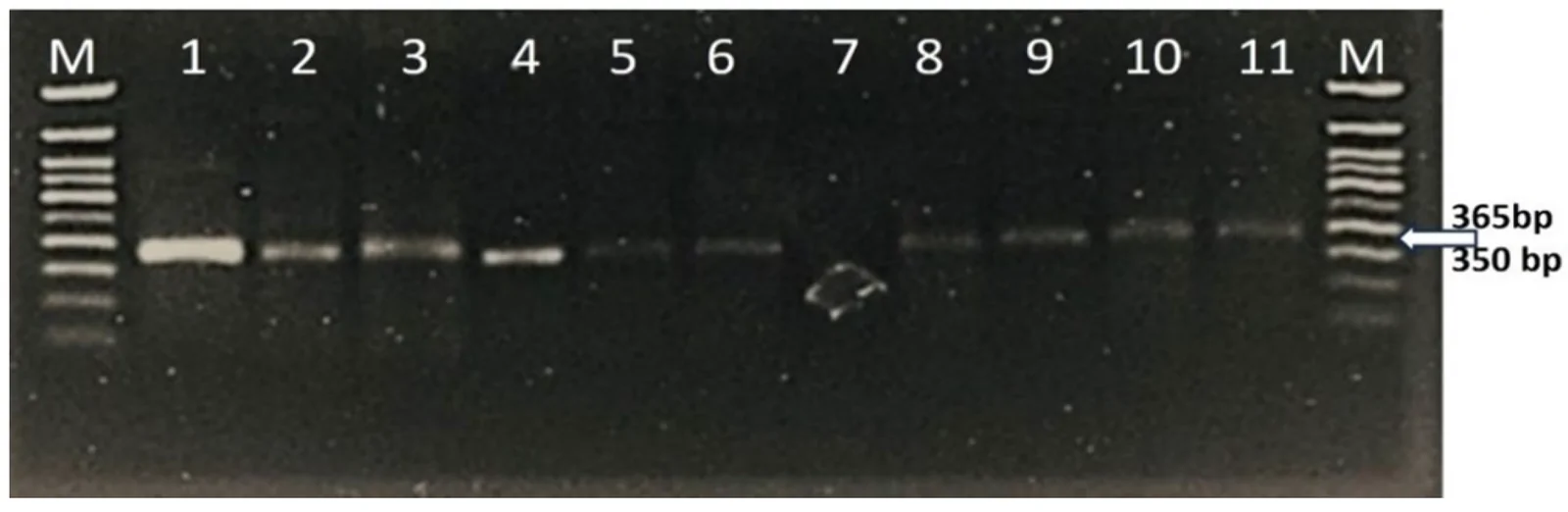
Agarose gel (2%) electrophoresis showing RT-PCR amplified products of four samples (lanes 1, 2, 3, 8, 9, 10, 11), amplified from tomato with the size of approx. 364 bp and (lanes 4, 5, 6) amplified from pepper plants with the size of approx. 350 bp using a pair of universal primers, Pospi1-RE and Pospi1-FW. Lane 4 negative control. Lane M represents the PCR Sizer 100 bp DNA Ladder (Norgen Biotek Corp).

**Figure 4:**
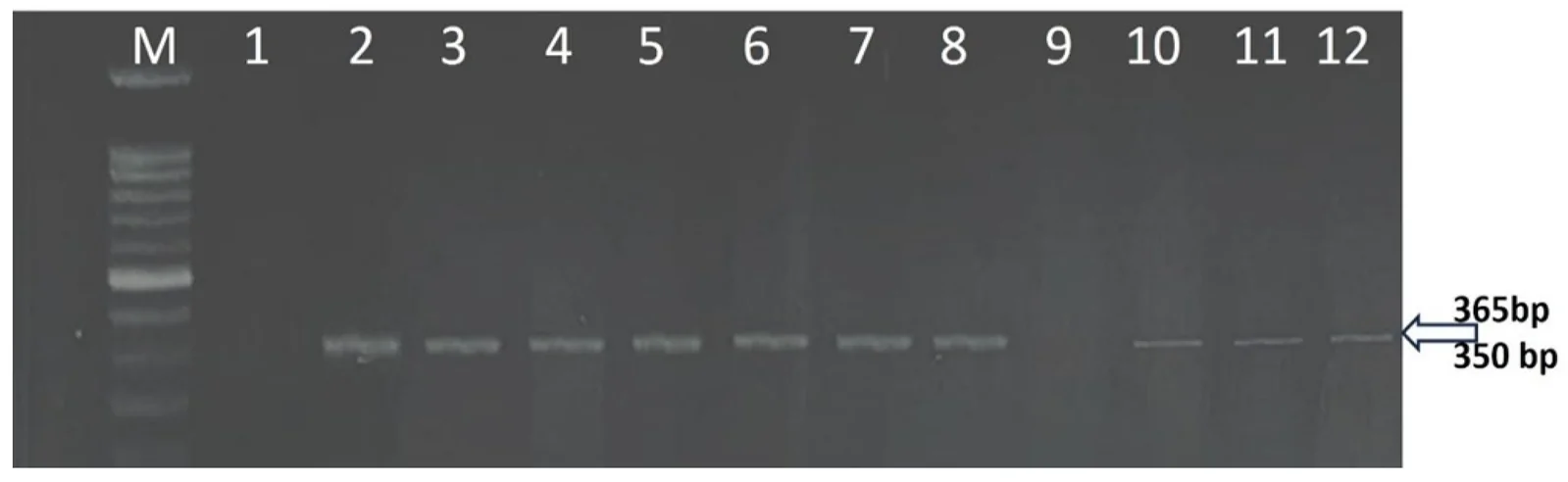
Agarose gel (2%) electrophoresis showing RT-PCR amplified products of four samples (lanes 2, 3, 4, 5, 6, 7, 8), amplified from tomato with the size of approx. 364 bp using specific primer for TASVd and (lanes 10, 11, 12) amplified from pepper plants with the size of approx. 350 bp using a pair of specific primers for PCFVd. Lane 1 and lane 9 as negative control. Lane M represents the GeneDirex (1KB) DNA Marker (GeneDireX, Inc.).

Out of the tested samples collected from tomato and pepper greenhouses, which were assayed with RT-PCR using the universal Pospiviroid primers, only six tomato samples and one pepper sample showed clear PCR amplification bands at the size of ~365 bp. Meanwhile, three pepper samples showed a PCR amplification band at the size equivalent to ~350 bp, which were collected from the Riyadh region (Al-Kharj area) only (Figure 3). Since the universal primers were used in this test, that confirms the presence of at least one of the nine Pospiviroid species that can be detected and amplified with these primers. On the other hand, having one sample with a band at a smaller size (approx. 350 bp) confirms the presence of one different Pospiviroid species compared to the other samples. The same bands of ~365 bp and ~350 bp were observed for these samples after the RT-PCR assay with the species-specific primers of TASVd and PCFVd respectively (Figure 4).

### 3.3. Sequencing and Phylogenetic Analysis

For TASVd, out of the seven RT-PCR products, that were sent for sequencing, two TASVd isolates from tomato and one from pepper had convenient sequences when these sequences were blasted in the NCBI website and showed high similarity to specific viroid species registered in GenBank. Three selected sequences isolates were then submitted to GenBank and designated as SA2 and SA4, which were identical to TASVd and were collected from tomato plants, and SA5, which was also identical to TASVd and was collected from a naturally infected pepper plant; they had the following accession numbers: MK286920, MK286921 and MK286922 respectively.

Furthermore, the obtained results showed that the nucleotide sequence of the Saudi isolate that was identical to PCFVd was collected from a pepper plant and is highly similar to all PCFVd isolates that were submitted to GenBank. This isolate was designated as SA7 and submitted to GenBank under the accession no. MK286923.

When comparing the Saudi isolates of TASVd with each other, isolate SA5 (that was collected from a pepper plant) had the highest similarity with the SA2 and SA4 isolates (that was collected from a tomato plant) grown in the same greenhouse (98.3%). The high similarity between the two isolates, SA2 and SA5, which were collected from different plant species (tomato and pepper), strengthened the probability that the initial source of infection came from tomato seeds and was mechanically transmitted to pepper plants during the growing season, as discussed earlier.

**Figure 5:**
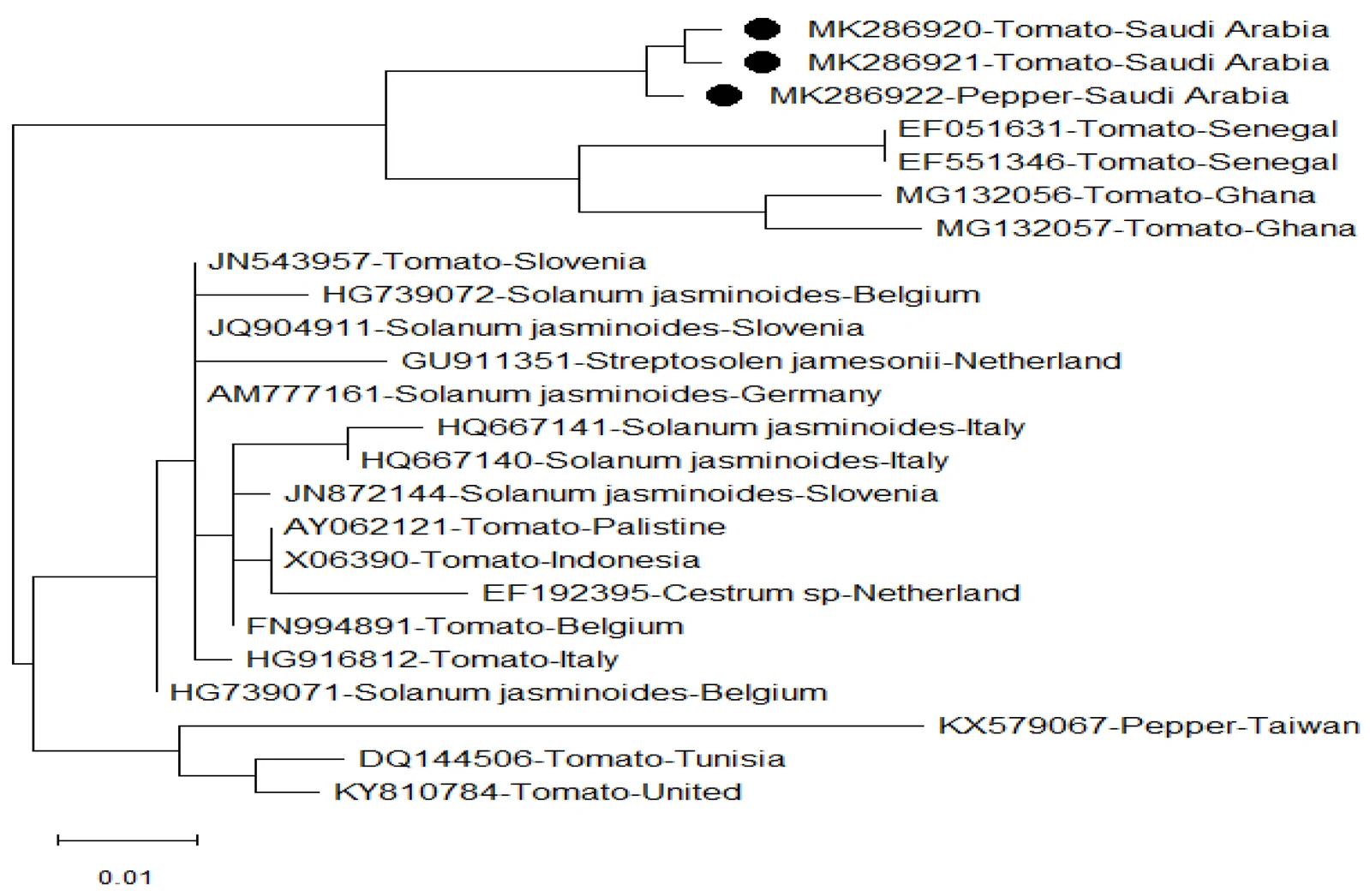
Homology tree based on multiple sequence alignment between three isolates of TASVd from Saudi Arabia and produced with 21 different TASVd isolates of the same species from different countries obtained from GenBank. Included is a figure showing how the colors and pairwise identities correspond.

**Figure 6:**
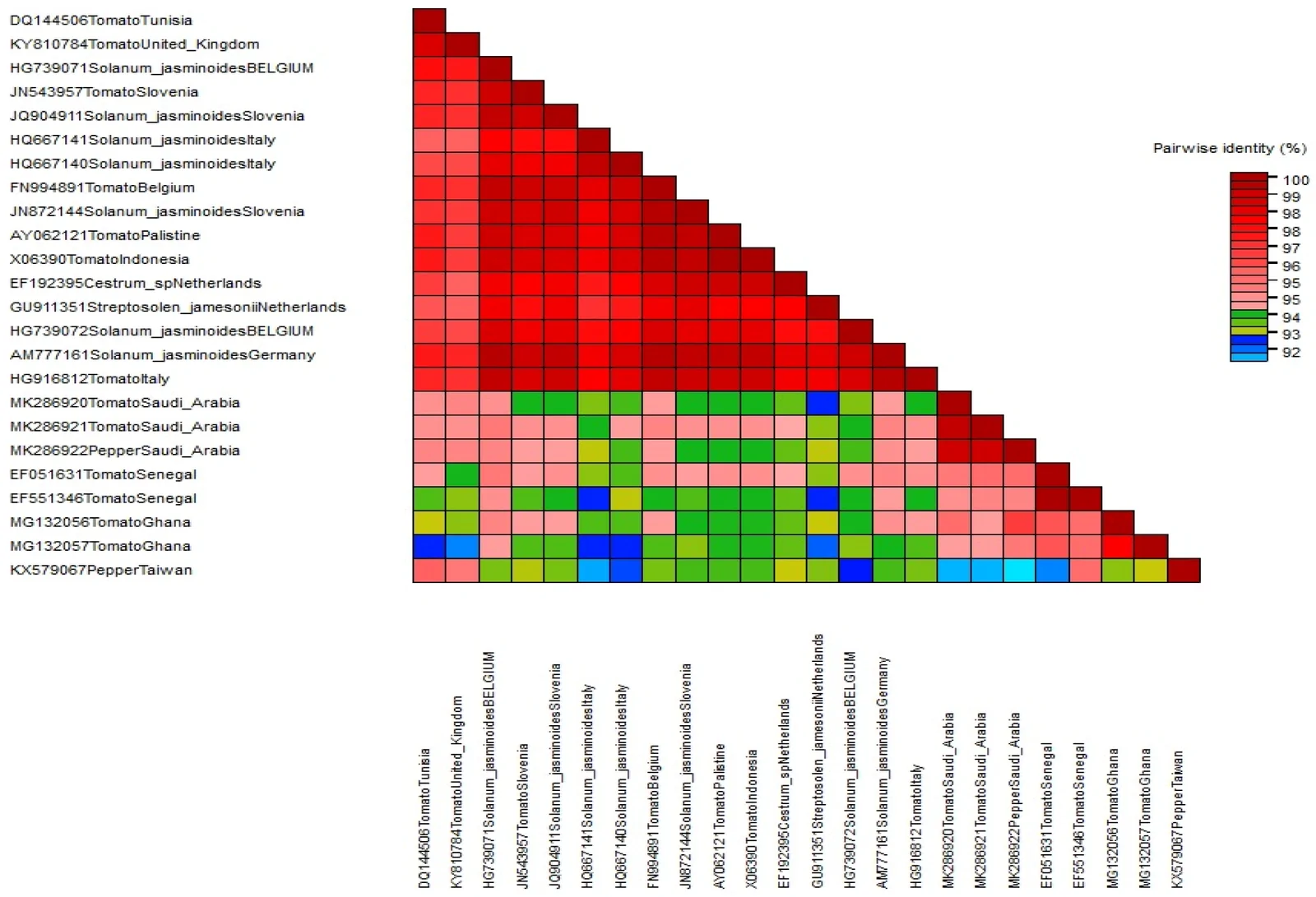
SDT matrix of pairwise identity scores produced with 21 different TASVd isolates of the same species from different countries obtained from GenBank. Included is a figure showing how the colors and pairwise identities correspond.

The TASVd isolates from Saudi Arabia had the highest similarity (93–95.7%) with the isolates from Ghana (MG132056 and MG132057) and Senegal (EF051631 and EF551346) and the lowest similarity (91.3–92.7%) with isolate GU911351 from the Netherlands and isolate KX579067 from Taiwan. The genetic relationship and homology tree for all Saudi isolates of TASVd are shown in Figure 5 and Figure 6 and Table 1.

**Figure 7:**
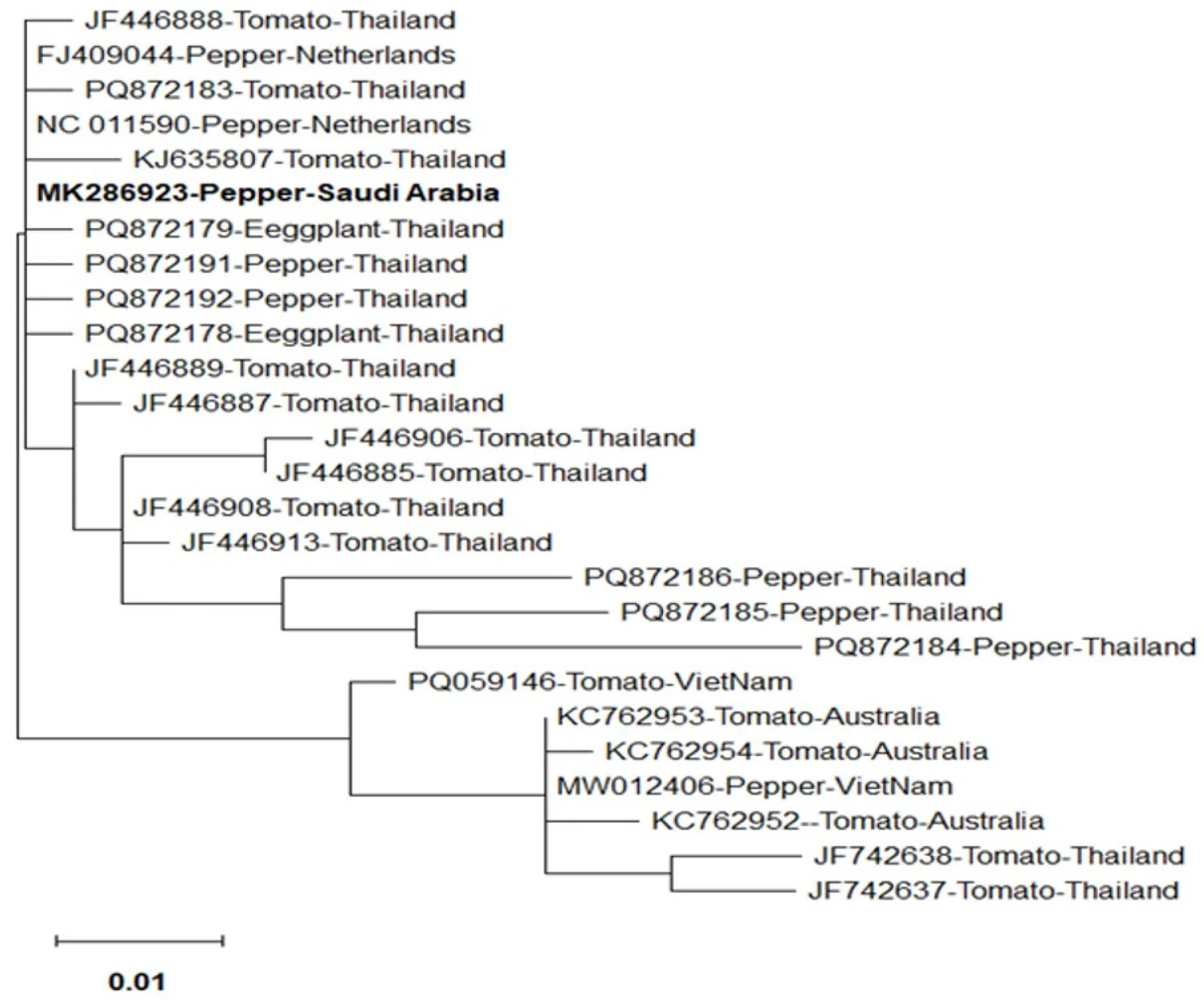
Homology tree based on multiple sequence alignment between three isolates of PCFVd from Saudi Arabia produced with 25 different PCFVd isolates of the same species from different countries obtained from GenBank. Included is a figure showing how the colors and pairwise identities correspond.

**Figure 8:**
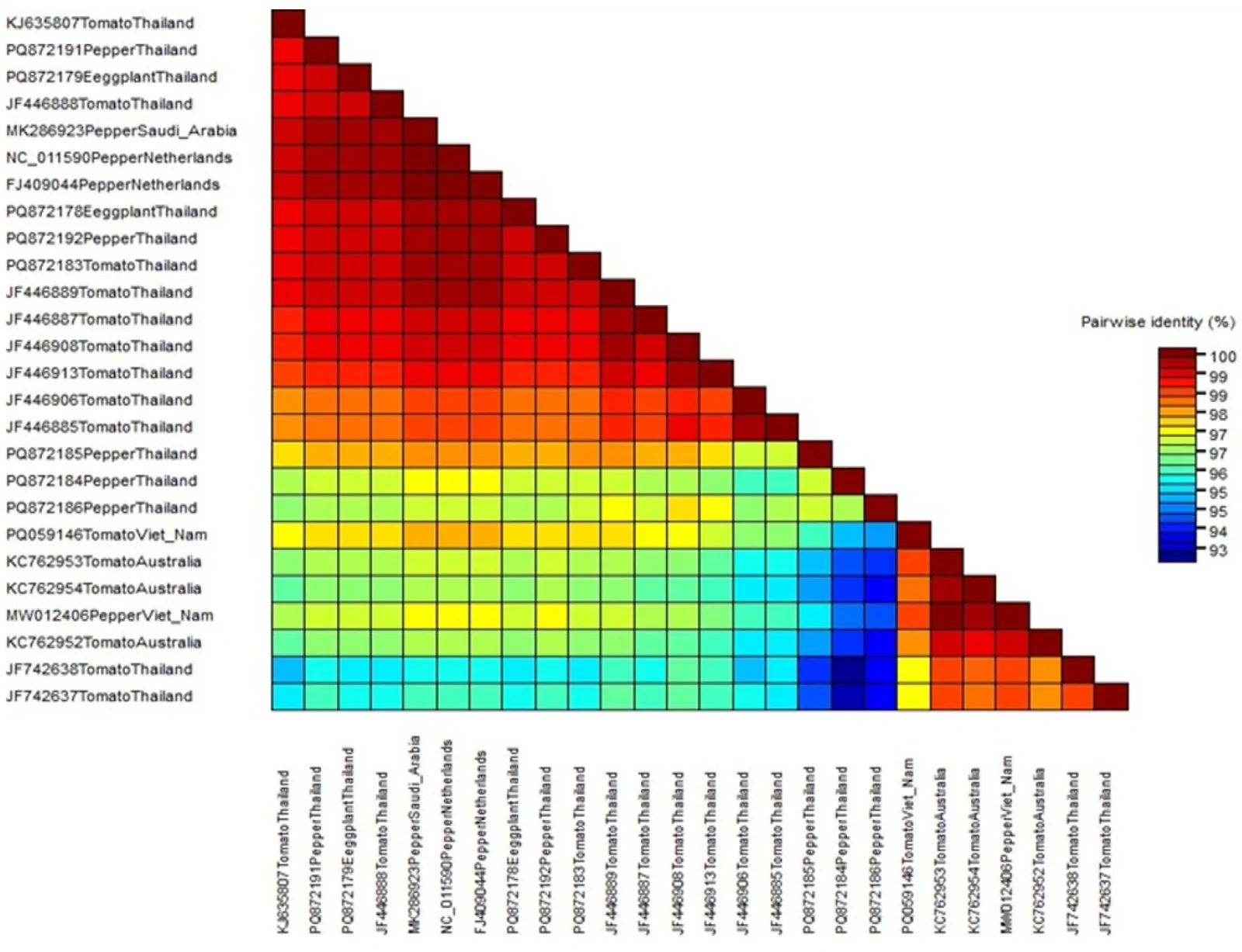
SDT matrix of pairwise identity scores produced with 25 different PCFVd isolates of the same species from different countries obtained from GenBank. Included is a figure showing how the colors and pairwise identities correspond.

The Saudi isolate of PCFVd, which was collected from pepper plants, had the highest similarity (100%) with the isolate no. FJ409044 collected from sweet pepper (*C. annum*) in the Netherlands [16]. The Saudi isolate was also highly similar (>99%) to many other isolates from tomato plants collected from different locations in Thailand, Australia and Vietnam. The genetic relationship and homology tree for the Saudi isolate of PCFVd is shown in Figure 7 and Figure 8 and Table 2.

### 3.4. Tomato Cultivar Responses

**Figure 9:**
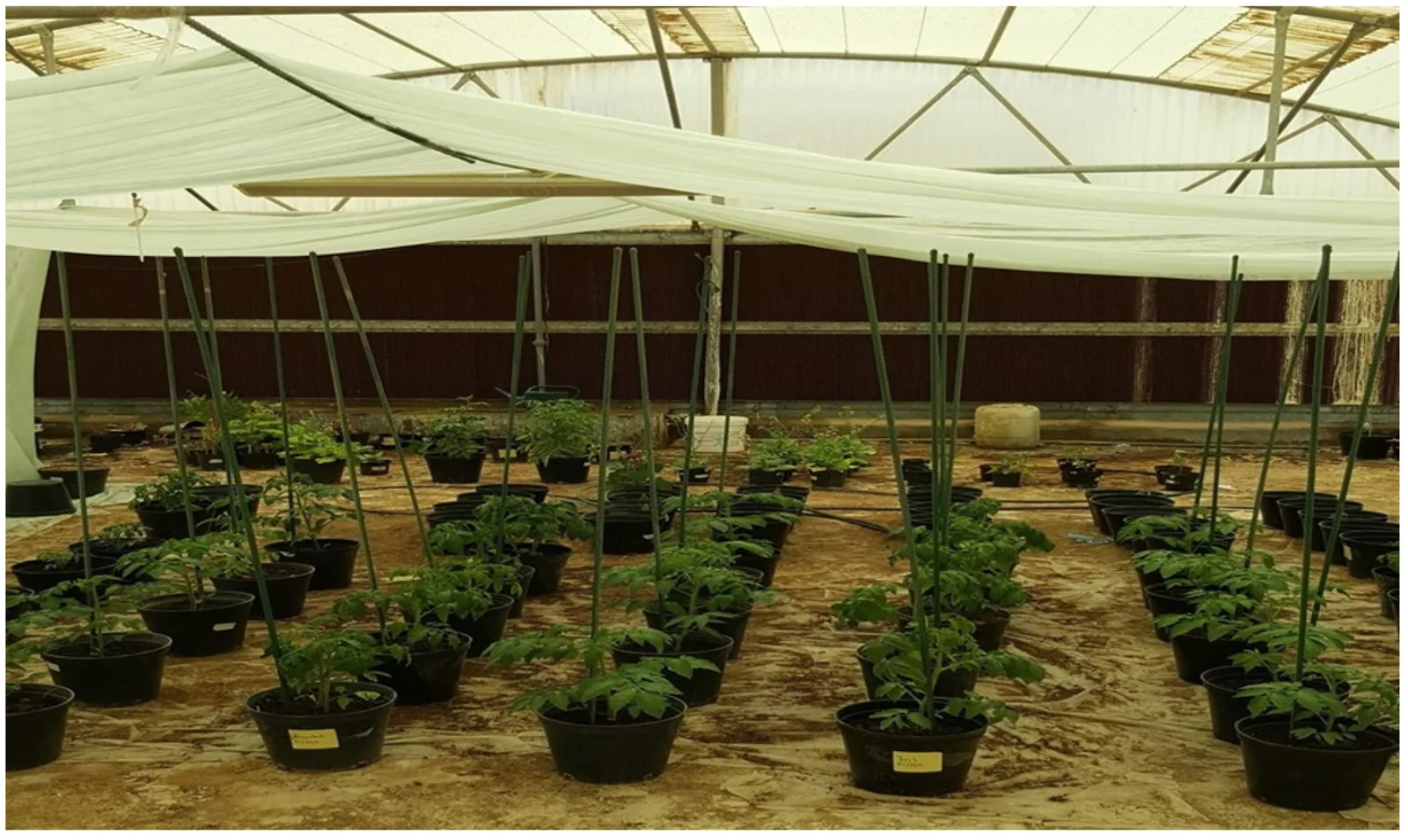
Tomato cultivar responses to mechanical inoculation with TASVd-isolate in the first greenhouse experiment.

**Figure 10:**
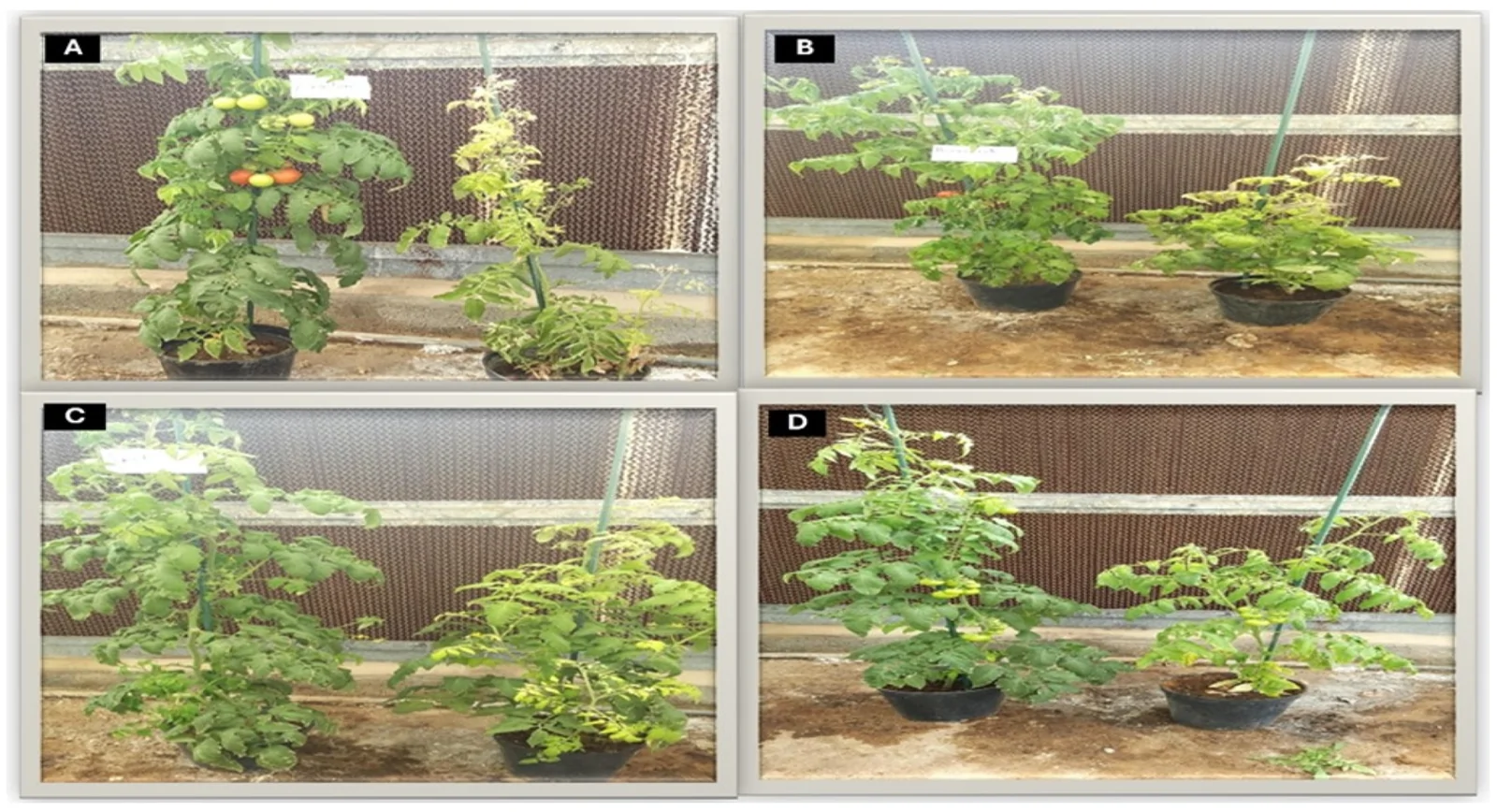
The effect of mechanical inoculation with TASVd on Newton (**A**), Beisuzhok (**B**), Irit (**C**) and Alindi (**D**) tomato cultivars (to the right) compared with the control plant (to the left).

In this experiment, the first symptoms on tomato plants of all cultivars appeared at least one month after inoculation with the local isolate of TASVd (Figure 9 and Figure 10). All inoculated cultivars responded with significant reductions in plant height when compared with the non-inoculated plants, which varied from 21.8–26.9%. Meanwhile, when comparing the percentage of reduction between the different cultivars, no significant differences were observed in the plant heights of all cultivars because of their mechanical inoculation with TASVd (Table 3).

Yet it was observed that all cultivars severely affected fruit weight. When the four cultivars were compared based on the effect of TASVd on their fruit weights, clearly significant differences were observed between them, and the Newton cultivar was the most affected, with a 90.2% reduction in fruit weight, while the Irit cultivar was the least affected, with 49.1% reduction (Table 4).

Dry weights of infected plants were significantly reduced when compared with the control plants, except for the Alindi cultivar. When comparing the reduction percentages of plant vegetative dry weight of the four cultivars, the Newton cultivar, with the highest reduction percentage in dry matter (39.3%), was the only significant one compared to the other three cultivars (Table 5).

The studied cultivars’ plant heights did not differ significantly from one another. The greatest decrease in vegetative dry weight (39.3%) was observed in the Newton cultivar. With a just 6% decrease in comparison to their esteemed health controls, the Alindi cultivar was the least impacted. With a 90.2% decrease in fruit output when compared to its untreated control, the Newton cultivar was the most impacted cultivar. However, with a 49.1% decrease in fruit output, the Irit cultivar was the least impacted.

## 4. Discussion

Viroid diseases, especially those of fruit and vegetable crops, occur widely in Middle Eastern and African countries, where the expression of viroid disease symptoms increases because of the hot or warm climates of these regions. Natural viroid infections belong to several members of the Avsunviroidae and Pospiviroidae families. Tomato apical stunt disease was initially reported in 1980 [26] from tomatoes in the northern areas of the Ivory Coast of Africa (Cote D’Ivoire), and the causal agent, TASVd, was the first viroid to be characterized on the African continent. Since then, TASVd has been reported in asymptomatic ornamentals and tomato in different parts of Africa [21,27,28,29], Asia [30,31,32,33] and various European countries [34,35,36,37,38,39].

The symptoms recorded in this study were like those described by Matthews-Berry, in 2010 [40], in the UK on tomato. Fruits of infected plants were smaller, ripped earlier, and had a pale red discoloration and early ripening. In severe cases, mummification was observed. Similar symptoms on fruits have been described [21,26,27,35,41]. However, these symptoms were less severe in PCFVd infected plants compared to TASVd infection. Similar symptoms were described earlier in pepper plants infected with PCFVd in the Netherlands [16]. However, leaf curling, which was observed in this study, was not reported in Verhoeven’s study. On the other hand, it should be mentioned that this is the first study ever that reports a natural infection of TASVd in pepper in the world; no symptoms were described for this viroid on pepper plants beforehand. We cannot distinguish between the infection of the two species (TASVd and PCFVd) on pepper plants based on the observed symptoms. Similar symptoms produced by different species of the genus Pospoviroid on the same host were recorded in studies earlier [13,14,22,42,43]. Moreover, after first being identified in Côte d’Ivoire, TASVd was later found in Indonesia, Israel, Tunisia, Senegal, Ghana, and other European nations. Reports from Senegal and Ghana, where TASVd was found in tomato crops, and Israel, where TASVd caused severe disease in greenhouse-grown tomatoes, are especially pertinent to the current investigation. The experimental host range of the Israeli TASVd strain, which was spread through mechanical inoculation and grafting, was different from that of the original Côte d’Ivoire strain, indicating that biological characteristics can differ amongst TASVd isolates [21,30].

Molecular detection methods for TASVd, and for viroids in general [44,45,46,47], include nucleic acid hybridization [48,49], RT-PCR [50,51,52,53], real-time RT-PCR [50,51] and next-generation sequencing [54]. Hence, in the present study, RT-PCR amplification was employed for the molecular detection of TASVd and PCFVd. The obtained results showed that the same bands of ~364 bp and ~350 bp were observed for these samples after the RT-PCR assay, with the specific primers of TASVd and PCFVd respectively, like [19,20,21,22,23].

On the other hand, the distribution of infection of the two viroids in one planting area, among all other areas, suggests that the initial source of infection might be through seeds, and the spread of infection during the growing season was probably via mechanical means. This possibility can be strengthened by the fact that all positive tomato samples were collected from the Beisuzhok (Giverech) cultivar, which was widely grown in the Al-Kharj area at that time. The seed transmission of many Pospiviroid species, including TASVd, PSTVd and CEVd, was already reported in several previous studies [8,10]. Using the universal Pospi1 primer pair in the RT-PCR test, no Posiviroid species were found in the remaining examined samples. Finally, only the TASVd viroid has been confirmed to infect tomatoes, while TASVd and PCFVd have been confirmed to infect pepper plants in the tested samples.

Mechanical and graft transmission of TASVd have been shown in experimental investigations. Furthermore, an epidemiological investigation in Israeli greenhouse tomato crops showed that bumble bees could spread the viroid across tomato plants during pollination, but neither *Myzus persicae* nor *Bemisia tabaci* could spread TASVd. In that study, seed transmission was also demonstrated; in the experimental conditions, transmission rates were reported to reach 80% [33].

However, in the greenhouse experiment, one positive TASVd Saudi isolate was selected to evaluate the severity and symptom responses to mechanical inoculation of four tomato cultivars. According to the data obtained, each cultivar exhibited a significant change in each metric.

## 5. Conclusions

In conclusion, TASVd was found for the first time on greenhouse-growing tomato plants, whereas TASVd and PCFVd were also found for the first time on greenhouse- growing pepper plants in Saudi Arabia. This is the first report of TASVd infection of pepper plants in the world. The two viroids were isolated and identified from the Al-Kharj, area and none of the other tested samples collected from other planting areas were found to have any of the Pospiviroid species. According to the results obtained in this study, seeds might be the initial source of infection, and mechanical transmission may take place later throughout the season. All Pospiviroid-infected tomato plants found in this study were from the same cultivar, Beisuzhok (Giverech). TASVd infection on tomato plants can cause severe damage and yield reduction of up to 90% in some cultivars. Different tomato cultivars have different responses to TASVd infection, and those may significantly vary with damage and production losses due to infection. Some plant species might be symptomless carriers of TASVd. Further studies to other agricultural areas in the Kingdom of Saudi Arabia are recommended to check for the presence of any of the Pospiviroid species on greenhouse-grown solanaceous crops. Testing some of the imported tomatoes seeds is highly recommended to determine the initial source of infection. Quarantine legislations for testing of Pospiviroid species are recommended to prevent the possible entrance of any other Pospiviroid species to Saudi Arabia. Developing breeding programs to produce new tomato cultivars with resistance or high tolerance to Pospivroidae species infection, including TASVd, is recommended.

## Figures and Tables

**Table 1:** Comparison of percentages of nucleotide sequences between TASVd Saudi Arabian isolates and isolates originating in different countries.

Accession No.	Host	Isolate	Country	MK286920	MK286921	MK286922
MK286920	Tomato	Al-Kharj-SA2	Saudi Arabia	100	98.3	98.3
MK286921	Tomato	Al-Kharj-SA4	Saudi Arabia	98.3	100	98.3
MK286922	Pepper	Al-Kharj-SA5	Saudi Arabia	98.3	98.3	100
AM777161	*Solanum jasminoides*	Sj1	Germany	93.8	93.3	94.1
AY062121	Tomato	-	Palistine	93.5	93	93.5
DQ144506	Tomato	-	Tunisia	93.3	93	93.8
EF051631	Tomato	-	Senegal	94.7	95.2	95.2
EF192395	*Cestrum* sp	3123751	Netherlands	91	92.1	92.1
EF551346	Tomato	-	Senegal	94.1	94.1	94.7
FN994891	Tomato	Brug1	Belgium	93.8	93.3	94.1
GU911351	*Streptosolen jamesonii*	4730772	Netherlands	92.4	92.1	92.7
HG739071	*Solanum jasminoides*	11/0197/VI.2	Belgium	93.8	93.3	94.1
HG739072	*Solanum jasminoides*	11/0238/VI.1	Belgium	93	92.4	93.3
HG916812	Tomato	-	Italy	93.5	93	93.8
HQ667140	*Solanum jasminoides*	sj4	Italy	93.3	92.7	93.5
HQ667141	*Solanum jasminoides*	sj10	Italy	93.3	92.7	93.3
JN543957	Tomato	Sj 403-Slo	Slovenia	93.8	93.3	94.1
JN872144	*Solanum jasminoides*	Sj 464-Slo	Slovenia	93.8	93.3	94.1
JQ904911	*Solanum jasminoides*	Sj 499-Slo	Slovenia	93.8	93.3	94.1
KX579067	Pepper	5458774	Taiwan	91.6	91.3	91.9
KY810784	Tomato	FERA_130827	United Kingdom	93.8	93.5	94.1
MG132056	Tomato	Touboudan	Ghana	94.4	94.7	94.9
MG132057	Tomato	Akumadan	Ghana	93	93.3	93.5
X06390	Tomato	indonesian	Indonesia	93.3	92.7	93.3

**Table 2:** Comparison of percentages of nucleotide sequences of PCFVd Saudi Arabian isolates with isolates originating in different countries.

Accession No.	Host	Isolate	Country	MK286923
MK286923	Pepper	Al-Kharj-SA7	Saudi Arabia	100
FJ409044	Pepper	-	Netherlands	100
NC_011590	Pepper	-	Netherlands	100
JF446885	Tomato	CR11-15c2	Thailand	98.6
JF446887	Tomato	CR14-8c5	Thailand	99.4
JF446888	Tomato	CR14-9c2	Thailand	99.7
JF446889	Tomato	LPng19-3c1	Thailand	99.7
JF446906	Tomato	LP1-15c2	Thailand	98.6
JF446908	Tomato	LP2-5c2	Thailand	99.4
JF446913	Tomato	LP4-8c1	Thailand	99.1
JF742637	Tomato	Aurai 2	Thailand	95.1
JF742638	Tomato	Aurai 10	Thailand	95.1
KC762952	Tomato	EMAI12/80420	Australia	96.3
KC762953	Tomato	EMAI12/759	Australia	96.6
KC762954	Tomato	EMAI12/761	Australia	96.3
KJ635807	Tomato	NP6-10c2	Thailand	99.4
MW012406	Pepper	PCFVd_NBHP	Vietnam	96.6
PQ059146	Tomato	PCFVd_TG	Vietnam	97.7
PQ872178	Eggplant	SB_Eg_PC43	Thailand	97.7
PQ872179	Eggplant	B_Eg_PC53	Thailand	97.7
PQ872183	Tomato	SB_Pe_PC31	Thailand	99.7
PQ872184	Pepper	SB_Pe_PC1	Thailand	97.1
PQ872185	Pepper	SB_Pe_PC3	Thailand	98
PQ872186	Pepper	SB_Pe_PC5	Thailand	96.8
PQ872191	Pepper	SB_Pe_PC13	Thailand	99.7
PQ872192	Pepper	SB_Pe_PC16	Thailand	99.7

**Table 3:** The impact of inoculating four different tomato cultivars with TASVd (isolate 312). Plant height means (in centimeters) and a comparison of the percentage decrease in tomato cultivars produced in greenhouses.

* Cultivar	** Control	** Inoculated	*** Reduction (%)
Newton	120 ^a^	94 ^b^	21.8% ^a^
Alindi	130 ^a^	95 ^b^	26.9% ^a^
Irit	131 ^a^	100 ^b^	23.7% ^a^
Beisuzhok	119 ^a^	92 ^b^	22.7% ^a^

* For each treatment, five plants were used per cultivar, and data were recorded at 90 days post-inoculation. ** For each cultivar, means followed by the same letter in the control and inoculated columns are not significantly different (*p* = 0.05) according to the LSD test. *** Average percent reductions in the cultivars followed by the same letter in the percent reduction column are not significantly different (*p* = 0.05) according to the LSD test.

**Table 4:** Effect of TASVd inoculation (isolate 312) on different tomato cultivars. Means of fruit weight (g/plant) and comparison of the percent reduction in four greenhouse-grown tomato cultivars inoculated with the local isolate of tomato apical stunt viroid (TASVd).

* Cultivar	** Control	** Inoculated	*** Reduction (%)
Newton	320 ^a^	31 ^b^	90.2% ^a^
Alindi	353 ^a^	97 ^b^	72.5% ^b^
Irit	417 ^a^	212 ^b^	49.1% ^d^
Beisuzhok	343 ^a^	136 ^b^	60.3% ^c^

* For each treatment, five plants were used per cultivar, and data were recorded 90 days after inoculation. ** For each cultivar, means followed by the same letter in the control and inoculated columns are not significantly different (*p* = 0.05) according to the LSD test. *** Average percent reductions in the cultivars followed by the same letter in the percent reduction column are not significantly different (*p* = 0.05) according to the LSD test.

**Table 5:** Effect of TASVd inoculation (isolate 312) on different tomato cultivars. Means of fruit weight (g/plant) and comparison of the percent reduction in four greenhouse-grown tomato cultivars.

* Cultivar	** Control	** Inoculated	*** Reduction (%)
Newton	112 ^a^	68 ^b^	39.3% ^a^
Alindi	119 ^a^	112 ^a^	6.0% ^b^
Irit	132 ^a^	116 ^b^	12.6% ^b^
Beisuzhok	114 ^a^	93 ^b^	18.1% ^ab^

* For each treatment, five plants were used per cultivar, and data were recorded 90 days after inoculation. ** For each cultivar, means followed by the same letter in the control and inoculated columns are not significantly different (*p* = 0.05) according to the LSD test. *** Average percent reductions in the cultivars followed by the same letter in the percent reduction column are not significantly different (*p* = 0.05) according to the LSD test.

## Data Availability

The genome sequence data of viroids presented in this study are available on the NCBI database through accession numbers MK286920, MK286921, MK286922 and MK286923.
